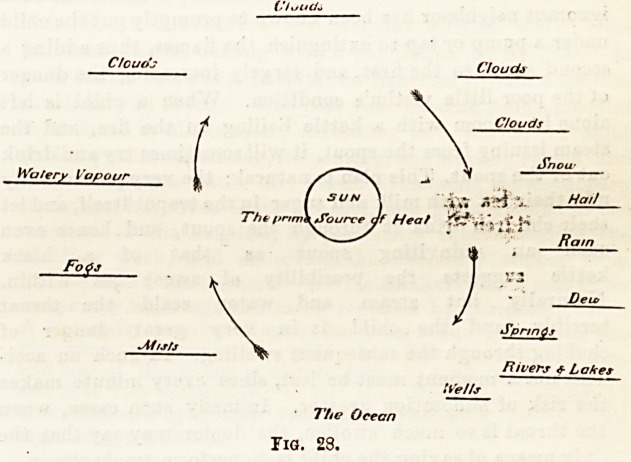# "The Hospital" Nursing Mirror

**Published:** 1896-07-04

**Authors:** 


"The Hospital" Nursing Mirror.

Being the Extra Nursing Supplement of "The Hospital" Newspaper.

[Contributions for this Supplement should be addressed to the Editor, The Hospital, 428, Strand, London, W.C., and should have the word "Nursing" plainly written in left-hand top corner of the envelope.]

### News from the Nursing World.

### THE QUEEN'S RECEPTION OF THE JUBILEE NURSES.

As we go to press some four hundred members of the Queen Victoria Jubilee Nursing Institute are enjoying their eagerly-expected day at Windsor. A full account of Thursday's proceedings will be given next week. The programme is as follows. The nurses meet at Paddington at 12.50, and go down by special train to Windsor, where they will find luncheon ready for them on their arrival in a tent in the park. Afterwards they will be shown over the castle, and then will form into a square to await the arrival of her Majesty. After the Queen has driven away tea will be provided for the nurses, who will then return to London to Grosvenor House, thrown open to them by permission of the Duke of Westminster. Madame Albani has promised to sing in the evening.

### PRINCESS MAUD'S WEDDING.

The Princess Maud's marriage is fixed for the end of this month, though the actual date has not yet been announced as we go to press. A replica of the tea-table, the Pension Fund Nurses' wedding gift, is still on view at 5, New Bond Street, by kind permission of Mr. Barker, the maker.

### QUEEN CHARLOTTE'S HOSPITAL.

The Duke of Cambridge paid a visit to Queen Charlotte's Lying-in Hospital on Wednesday in last week. His Royal Highness was received by Viscount Portman and members of the committee and medical staff. An address presented to him referred to the need for various alterations and improvements, and for a new nurses' home, requiring a sum of £7,000. The Duke afterwards went round the wards, and before leaving complimented the nursing staff on the general appearance of the wards, and the manner in which they appeared to do their work. He was glad to know that so many women were willing to take up such responsible duties.

### NEW NURSING HOME AT ADDENBROOKE'S.

The nursing staff at Addenbrooke's Hospital are rejoicing in their new and comfortable quarters. The training home just completed is at the back of the hospital buildings, and has sleeping accommodation for thirty-eight nurses. It has, therefore, been possible already to make a much needed increase in the staff. It is a three-storeyed building, the top floor being devoted to the night nurses. To minimise noise the corridors are floored with pitch pine blocks. Incandescent light is used, and heating is accomplished by hot-air pipes. The home is connected by a covered glass corridor with the nurses' "sick-room," familiarly known as "Paradise" amongst themselves, where cases of illness amongst the staff can be treated. This little nurses' hospital was built in 1890, and was furnished by the nurses. Towards the furnishing of the new home about £70 has been contributed by past and present probationers. The estimate for the whole building is £3,400, towards which Mr. A. Peckover, Lord Lieutenant of the county, has given £2,000.

### NURSING AT TOTNES WORKHOUSE INFIRMARY.

An important question has lately come up for consideration before the Totnes Board of Guardians. The nursing staff have resigned on finding that they were expected to undertake as part of their regular duties the receiving and bathing of vagrants. It is once more a case of economy in the wrong place. The saving of a few pounds is of far higher importance to the mind of the ordinary guardian than the insistance upon an adequate and efficient administration of the institution under their care. It is quite certain that the bathing of tramps is not a part of the work of the trained nurses in a poor law infirmary. It may prove a very dangerous channel of infection to the sick, especially in the case of a midwife, and is to be strongly deprecated as a practice. The cost of paying someone else to see to this duty would be very trifling. The nurses at Totnes were supplied through the Workhouse Infirmary Nursing Association, where the guardians have decided to apply again, explaining the "small number of tramps." We hope the Totnes Guardians will, in the result, see the wisdom of relieving their nurses of an unsuitable piece of work, and that this should be done without running counter to the strongly expressed opinion of the medical superintendent that the infirmary cannot be worked with a smaller staff of trained nurses.

### AN "EGG SERVICE."

A new idea in the way of flower services was carried out at the Mission Church of St. John, West Streatham, a Sunday or two ago, when, in addition to floral offerings, numbers of eggs were brought also, nestling in baskets of moss or covered with flowers, and painted with texts and mottoes. In all about 1,700 eggs were brought to the church, and after the service were promptly despatched to St. Thomas's Hospital.

### AT THE PEOPLE'S PALACE.

At the exhibition now being held at the People's Palace in the Mile End Road, nurses will find much to interest them in the "London Hospital stall," presided over by one of the sisters from the hospital. Here there are model beds and cots with beautifully bandaged doll occupants, specimens of splints, and dressings of all kinds, and a good show of preparations from the dispensary. There is a delightful "district nurse's cupboard," filled with nursing appliances and necessaries, and a new arrangement for giving an ice pack. From the Poplar Hospital also there are some contributions, notably the "Bloxham" splint, and an excellent ward lamp. Messrs. Down have a stand of instruments, and show some of their very up-to-date ward furniture. A series of nursing lectures is also being given on Tuesdays and Thursdays in one of the lecture halls, by Miss Tillyard, a late "London" sister. The exhibition itself is well worth even a pilgrimage from the other end of London on a hot day, there is so much to be seen that no one should miss. There is one exhibit which is quite unique. It is a stall of grotesque little figures in the shape of match-box holders, and other odd devices, most cleverly contrived with chicken bones and shells. The lady whose invention it is, shows great originality and talent, and her ideas are admirably executed.

### THE NORLAND INSTITUTE.

Inquiries reach us from time to time with regard to the Norland Institute. Perhaps a short statement of the objects for which it exists may be useful. The institute is in Holland Park Avenue, and here 18 to 20 ladies may be received at a time for training as children's nurses. The period of training extends over nine months, during the first three the students remaining in the institute and devoting their days to the study of Kindergarten methods, needlework, cutting out and making of children's garments, domestic duties, cookery, and so forth. Lectures are given on simple hygiene and useful knowledge. For the second three months the would-be nurse goes as a paying probationer to a hospital to gain a little rudimentary nursing knowledge, and the last three months are spent in a family, with the option of remaining if she gives satisfaction. There are many women seeking employment, and fond of children, to whom a congenial career is thus opened.

### BICYCLING FOR NURSES.

Two interesting letters appear in this month's *Nursing Notes* on the subject of bicycles for nurses. The writers are both Queen's Nurses, who speak from practical experience of the advantages of a bicycle for district work, one being Miss Saunders, lady superintendent of the Peterborough District Nursing Association. Useful hints as to dress may be gleaned therefrom, the uniform cloak being condemned as quite unsuitable. The Peterborough Committee gave the nurses a bicycle, a wise step, which has saved many a cab fare and much time. Miss Saunders computes that its use saved "in every day's work at least two hours," a weighty consideration for a busy district worker.

### IRISH MEDICAL STUDENTS AND A LADY EXAMINER.

Certain students of medicine in Ireland have raised objections to the appointment of Dr. Winifred Dixon as examiner in midwifery to the Irish Royal College of Surgeons, and laid their protest before the council. In reply they have been informed by the council that the appointment cannot be interfered with, having been ratified according to charter.

### A NEW NURSING HOME.

A new Nursing Home, Bulstrode House, Bulstrode Street, Cavendish Square, has been started by Lady Alice des Voeux, and was formally opened last week by the Duchess of Teck. A great number of people were present at the "At Home" given to celebrate the event, amongst whom were the Duchess of Abercorn, the Earl and Countess of Hardwicke, Earl and Countess of Cork, and Lord and Lady George Hamilton. The home has accommodation for eight patients, and one room contains three beds, divided by curtains, for the reception of patients at a somewhat reduced fee. The Duchess of Teck admired the arrangements generally, and the appearance of the nurses, who looked very neat in their blue linen dresses, white aprons, and mob caps. The home is in connection with one at Guildford in which Lady Alice also takes a keen interest.

### NEW INFIRMARY, ISLEWORTH.

The new infirmary, Isleworth, is to be opened by the Duchess of Teck at the end of July. This infirmary has been erected by the Brentford Guardians, and is a handsome building, containing twelve large wards, an operating theatre, and a separate building for the nurses' home, in which are nicely furnished rooms for the nursing staff, each sister, nurse, and probationer having a separate bed-room. There are a few vacancies for ward nurses who have had at least one year's experience in a general hospital. Probationers will receive a certificate at the end of a three years' course.

### WOMEN'S LIBERAL FEDERATION AND THE MIDWIVES REGISTRATION BILL.

The subject of the Midwives Registration Bill came up for discussion at the late meeting of the Women's Liberal Council. The following resolution was proposed by Mrs. Schwann, delegate for South Kensington: "That this Council desires to call the attention of Parliament to the urgent need for improvement in the position and education of midwives, and urges the Government to support the Bill for the Registration of Midwives which is now introduced in the House of Commons." Mr. Victor Horsley proposed an amendment substituting after the words "to support" "any practical scheme having for its object the registration of midwifery nurses, such nurses to be registered as midwifery nurses only, and to be under the supervision of medical practitioners." Dr. Annie McCall spoke strongly against the amendment, and in favour of the registration of midwives. The original resolution was finally carried almost unanimously.

### NURSING IN FRANCE.

Miss Entwistle, in the *Guardian* for June 10th, gives an interesting account of her visits to foreign hospitals, more especially last year to some of those in Paris. Having inspected the Hôtel Dieu and the Hospital of St. Louis, both nursed by devoted but "uninstructed" *religieuses*, Miss Entwistle visited the Hospital Beaujon, where the nursing is done by lay-women. Here things were even more behind the times, the wards dirty, and the nurses, in spite of being expected to follow a certain course of instruction to obtain a diploma, unable to take temperatures or even make poultices, while their knowledge on the subjects of ventilation and cleanliness were of the most elementary character. Matters were better at the Hospital St. Joseph, a modern pavilion building, nursed by the St. Vincent de Paul Sisters. Here the wards were clean and bright with flowers, and the nursing generally of a far more advanced character, the sisters taking temperatures and doing dressings. It is significant that this hospital is not under "L'Assistance Publique," but was built and is supported by private charity, and administered by a committee of ladies and gentlemen.

### DEVOTED SERVICE.

Medals of Honour have been awarded by the French Minister of War to two women for devoted service in connection with the late Madagascar Expedition. Madame Jay, better known as Sœur Zénaïde, the Superior of the St. Joseph de Cluny Mission, has worked in Madagascar for nearly fifteen years, and throughout the war devoted herself heart and soul to the care of the hospital at Majunga. The other recipient of the honour is an Englishwoman, Miss Christina Byam, who for thirteen years has filled the position of Directress at the English Hospital at Soavanandriana. The hospital during the war was used as an annexe to the military hospital at Antananarivo, and reserved especially for the reception of the sick and wounded among the French troops.

### Hygiene: For Nurses.

By JOHN GLAISTER, M.D., F.F.P.S.G., D.P.H.Camb., Professor of Forensic Medicine and Public Health, St. Mungo's College, Glasgow, &c.

### XIII. — WATER IN RELATION TO HEALTH. — SOURCES OF SUPPLY.—MODES OF SUPPLY.

WATER, like air and food, is one of the prime necessities of life, animal or vegetable. It enters into the composition of nearly everything in nature. Everyone is familiar with the disastrous effects of unsatisfied bodily thirst. The human body is made up of 75 per cent. of water, hence the daily need is not inconsiderable. Water is a potent factor also in personal, domestic, and public hygiene; hence the need for a plentiful supply to populous places which can be easily tapped by every consumer. The physical appearances of water are matters of common knowledge, but with its chemical composition most persons are less familiar. It is a chemical compound which results from the union of two gases, viz., hydrogen and oxygen. When two volumes of the former and one of the latter are mixed, and are exploded by an electric spark, a new liquid substance—water—is the result; therefore the chemical symbols for water are $H_2O$. Chemically pure water does not exist in nature, although it is found approximately pure. It exhibits interesting physical conditions upon occasion. It freezes from the effect of cold, and it boils from the effect of applied heat. Water may thus exist in one of three forms, viz., as a solid, below 32° Fahr., or 0° Cent., as a liquid between 32° and 212° Fahr., or 0° and 100° Cent., and as a gas, above 212° Fahr., or 100° Cent. When water in the gaseous form is reduced to the liquid form by cold the process is called condensation. This is a very important, and, for us, an absolutely essential operation of nature. When, again, water is cooled to the solid state of ice, a very interesting phenomenon is to be noted in the process of cooling. When the temperature falls to 39·2° Fahr., or 4° Cent., the process of contraction which the water has undergone between 212° and 39·2° is suddenly changed. From 39·2° it begins to expand, until the freezing point—32° Fahr.—is reached. Were it not for this fact, intense frost would convert our rivers and lakes into solid masses of ice, instead of a top layer of ice and a lower layer of water, as the foregoing condition demands. When water passes from the liquid to the solid state it expands, that is to say, the volume of ice formed is greater than the volume of water from which it was formed. It is erroneously believed by many that frozen pipes burst when the thaw comes. The fact, however, is that the pipe is burst by the expansion of the ice, but the fracture is not apparent till water can once more flow in the pipe, then the leak shows the fracture.

The boiling of water is also an interesting phenomenon. Water contains in solution the gases of the atmosphere, much in the same way as in aerated waters. The noisy, bubbling process, known as boiling, is simply the disengagement of these gases by the heat. After they have been driven off a noiseless boiling will ensue, if the heating be continued. The temperature at which water boils is always the same under the same conditions. The boiling point of water is always reckoned at a pressure of one atmosphere—that is to say, the pressure which a column of atmosphere, of assumed equal density and five miles in height, exercises on a body at sea level. If this pressure be increased or diminished the boiling point will rise or fall correspondingly. For example, water boils at the level of the Dead Sea at 214° Fahr., because that is about 1,300 feet below the ordinary sea level; on the other hand, at the top of Mont Blanc it boils at 186° Fahr., because that mountain is about 15,000 feet above sea level. The period of time required for cooking any article of food which demands boiling is reckoned by our experience of the fact at or near the sea level. But potatoes, for instance, will not be more quickly cooked at the top of Mont Blanc than in London or Glasgow. Cooking under increased or diminished pressure is now frequently carried out on the large scale. Papin's Digester—an apparatus largely used in Continental hospitals for making beef tea—is worked on the principle of increasing the pressure within the apparatus by placing weights on the air-tight lid. On the other hand, in public works where food stuffs and condiments are made, the cooking takes place in vacuum pans, in which the atmospheric pressure is diminished.

The public health largely depends upon the abundant supply and free use of wholesome water. A water famine is disastrous to the health of a city, as it also is to public economics. Where the carriage of sewage depends on the water supply, reduced consumption of water means impurity of sewage channels, and the risks to health which are consequent. But however ample a public water supply may be, experience has shown that unless it be easily accessible to every householder, it is not so freely used as it ought to be for cleansing purposes. The effect of this in poorer districts is certain to be harmful sooner or later.

SOURCES OF SUPPLY.—The original source of all the water

FIG. 28.

used by man at any point on the earth's surface is the ocean of water which encircles the land. Whether the proximate source of supply be lake or river, reservoir, rainfall, well, or spring, the original source is the same. Nature is at once a huge evaporating and condensing apparatus. Just as the sea is the storehouse of summer heat, which it gives back to us in the winter, so is it the source of the watery vapour which is driven from it by the sun into the atmosphere, which is condensed in the form of clouds, and which returns to us as water, either in a liquid or solid form. Fig. 28 is a diagrammatic representation of the process of evaporation and condensation. In the centre is the source of heat; at the bottom, the source of watery vapour; the ascending arrows indicate the ascent of this vapour, as mists, fogs, and on top of all, as clouds; the descending arrows, the descent of the vapour, as snow, hail, rain, dew, to form springs, rivers, lakes, and wells. Water, in one form or other, having fallen upon the surface of the earth, it depends upon the configuration of the land upon which it falls, and upon its geological structure, whether it will sink mainly into the earth or collect upon its surface. There is thus formed an underground or subsoil collection of water, and an overground or surface collection; and the level of each at any given time depends upon the antecedent rainfall. A well taps this underground supply, and the depth of the well before water is reached depends upon the distance from the ground surface the level of the underground water is. In some parts it is near, and in others far from the earth surface. That part of the rainfall which does not percolate into the soil forms lakes and rivers, the incidence and position of which depend upon the topography of the land and its geological formation. Springs arise from the underground water, which in the course of percolation downwards encounters an impervious rock stratum. This stratum dips out on the land surface, and the water travelling along it thus eventually appears as a spring.

### Trained Nurses' Clinic.

### VIII.—BURNS AND SCALDS.

Of all terrible accidents a severe burn or scald is one of the most dreadful, and such happen most frequently to young children or old people. How often does the hard-working mother have to leave behind her two little children, the elder one, itself a mere babe, to take care of the younger, whilst she herself goes out to get some necessary of life; and how often do these children get into trouble during even a short absence. Hospital experience shows that the young children of poor people often get burnt because their mothers tie them into high chairs by the side of a fire, vainly hoping they will thus be kept safe. As a matter of fact, however, a child so left alone at once becomes restless, and finally succeeds in upsetting itself and chair on to the fire.

Older children of all classes when left alone in cold weather frequently yield to the dangerous fascination of fire, play with it, and set their clothes alight; then, thoroughly alarmed, they scream and rush out of doors. A kind-hearted ignorant neighbour has been known to promptly put the child under a pump or tap to extinguish the flames, thus adding a second shock to the first, and largely increasing the danger of the poor little victim's condition. When a child is left alone in a room with a kettle boiling on the fire, and the steam issuing from the spout, it will sometimes try and drink out of the spout. This plan is natural; the very poor usually mix their tea with milk and sugar in the teapot itself, and let their children drink it through the spout, and hence even such an uninviting spout as that of a black kettle suggests the possibility of sweet tea within. Naturally hot steam and water scald the throat terribly, and the child is in very great danger of choking through the subsequent swelling. In such an accident not a moment must be lost, since every minute makes the risk of suffocation greater. In many such cases, when the throat is so much swollen, the doctor may say that the only means of saving the child is to perform tracheotomy.

Such scalds are so common in large towns, the danger is so great and imminent, that any nurse who has to do with them should always explain their urgency to parents and beg them to allow the doctor to do whatever he thinks best, and most likely to save the child. If this kind of accident happens in an out-of-the-way place, and the doctor lives at a great distance, he must be sent for without delay, and the messenger instructed as to what has happened. We have heard many country doctors complain that they could not learn details nor get any idea as to the help required, and have, therefore, arrived on the scene unprovided with the most needful things such as they could have brought had they only been furnished with a little more information.

To allay the terror of the patient and his friends is no mean part of the nurse's duty, and it is one of the first which she has to consider. If she succeeds in getting the child comparatively calm before the doctor's arrival it will make the latter's examination of him both easier and better.

Burns vary very much in seriousness according to the part of body, and the extent of surface which is injured. Thus a burn on the trunk is generally worse than one of equal extent and depth on the limbs, but a burn involving the whole of the lower limbs may be more serious than a burn on the trunk. The first danger in burn cases is due to what is technically called "shock," that is the effect on the nervous system of the destruction of or injury to a large number of nerves. Hence the reason why an extensive burn may be more serious than a small deep one. This "shock" to the system is what must be guarded against or counteracted as far as possible. After a burn a person will in all probability be cold and pale, perhaps even half unconscious. Hence the first thing to do is to try and restore warmth by all possible means. In all examples of severe burns or scalds it is well to impress on everyone concerned that the patient's condition must be attended to first, and the wounds second. Otherwise, it will be found that discussions on the best available dressings, or on the amount of clothes which must be cut away from the injured surfaces, prove of such engrossing interest that the sufferer's immediate critical condition is overlooked, and valuable moments are lost.

Of the various methods of dressing these wounds all trained nurses have usually had large experience, but, in giving instructions to their juniors, they can never be too careful in insisting on the necessity of unceasing tenderness and care in carrying out treatment. The cases are tedious ones, and even after they have safely passed through the first stage of danger, there remain weeks during which bad burn cases come to be looked on as very monotonous, and, indeed, reckoned almost as amongst the "old chronics."

It is small wonder that a person grows irritable under the trial of past and the anticipation of future dressings, which must always be accompanied by more or less pain. It is often the duty of the nurse to remind the friends of such sufferers of this fact, for a daily dressing comes to be such a matter of routine that it cannot but tend to some amount of indifference in those who are ignorant of the trial, and perhaps grow a little wearied of the patient's "unreasonableness." So much can be done by the nurse to lighten the burden of the patient's pain and by care of his general health, by consideration of any small ways or whims, that the possibilities of her gracious ministrations are infinite. The helplessness caused by an accident adds largely to the trial of the catastrophe. The sudden arrest of daily work, of active exercise and amusement, can be more philosophically borne than the minor discomforts which make themselves felt by degrees.

When the first anguish of body and distress of mind are allayed there follows the humiliation of complete dependence on others. To the self-reliant, reserved man and woman it is indescribably annoying to be thus cut off from the care of their own persons and possessions. Whilst the body lies helpless, the mind may be abnormally active, and the recollection of unfinished work on the littered desk, of open drawers hitherto closely guarded from prying eyes, of the overfull and not too tidy work basket, all these memories are present with the eager worker. The day entered on in perfect health has been turned in a moment by some unavoidable accident into one of horror, pain, and helplessness.

This is a side of the subject which should, and does, affect the demeanour of many a nurse, and aids her to bear with infinite patience the restless discontent, inconsiderateness, and impatience of persons who, unaccustomed to illness, rebel against the ill-luck which has reduced them for the time to the helpless dependence of children. On the nurse herself rests the responsibility of reconciling her charges to the changed condition of things, and to her credit, be it said, she seldom fails to do so.

### The Nightingale Home, St. Thomas's Hospital.

### "AT HOME," AND PRESENTATION TO MISS CROSSLAND.

THE annual "At Home," held by the Secretary to the Nightingale Fund (Mr. Henry Bonham Carter) and Mrs. Bonham Carter at St. Thomas's Hospital on June 25th, was this year the occasion chosen for a presentation to Miss Crossland, for twenty-one years home sister of the Nightingale Home, on her retirement from the post she has filled for so long, and in which she has gained universal love and esteem. Most of the expected guests had arrived by half-past five, and the presentation of a purse and an album, in which the names of the subscribing past and present Nightingale probationers (to the number of 273) were contained, was then made in the dining-room of the home by Mr. Croft, one of the consulting surgeons to the hospital, who had been associated with the training school for many years as medical teacher and attendant. Miss Crossland made an excellent little speech, which was greeted with much applause and appreciation, and afterwards Mr. Bonham Carter presented her with a bouquet from Miss Nightingale, with a card inscribed :—

To Miss Crossland, home sister of the Nightingale Home for twenty-one years, who has given a new life and calling to so many in nursing the poor, body and soul, by absolute self-devotion, training in wise discipline, loyalty, and love, as well as in technical and intellectual skill and knowledge.—A grateful, FLORENCE NIGHTINGALE.

The address inside the album read as follows :—

Dear Miss Crossland,—We, the undersigned past and present probationers in the Nightingale Training School, being desirous on the occasion of your retiring after twenty-one years' service, of presenting you with some small token of our esteem and affectionate regard, have joined in providing a purse, which we now offer for your kind acceptance, together with this book, in which our names are inscribed. We feel that we owe much to the principles instilled into us, and to the aid afforded to us by you during our probationary year, and many of us also for your kind advice and sympathy in our subsequent career, and we also feel sure that your example and your work have largely contributed to the creation and maintenance of that high standard of excellence in our common vocation to the attainment of which the life-long efforts of our chief, Florence Nightingale, have been directed.—We are, dear Miss Crossland, your affectionate friends.

The little ceremony over, Miss Crossland received many warm congratulations and heartily expressed regrets at the severence of her long connection with the home, where her ready sympathy and wise counsels will be greatly missed. The guests, amongst whom were the Bishop of Rochester and the Hon. Mrs. Talbot, the members of the committee, Sir William and Lady McCormac, Dr. and Mrs. Payne, Dr. and Mrs. Cullingworth, Dr. Sharkey, Mr. Clutton, and other members of the medical and surgical staff of the hospital, besides many matrons, and past and present Nightingale nurses and their friends, then dispersed into the grounds and on to the charming terrace. Fortunately, after a wet morning, the afternoon was brilliantly fine, and the pleasant gathering was fully as successful as usual, and much enjoyed by all who were present.

### "The Power for the Children."

EVERY grown man and woman should read the pamphlet thus headed, being the annual report of the National Society for the Prevention of Cruelty to Children, and reading it, should contribute something, be it ever so small, to the funds for which it pleads. During the seven years of its existence this society has worked marvels, and it is to-day the great power for the protection of children in the land. For of what use are legal enactments for their benefit if there are no champions to take up their cause and make their cries heard, as they are powerless to do for themselves? The horrors inflicted upon little children, and their helplessness, are such that one could hardly bear to think of it were it not for the splendid work which is being done through the instrumentality of this society. During the past year it has reached 52,871 children; in the name of the law 14,687 warnings have been given, 2,107 prosecutions instituted (of which the percentage of convictions was over 96½); and 38,407 supervision visits paid to warned persons and ex-prisoners. There is no form of rescue work which appeals so strongly to every humane person as that which saves little children from cruelty and misery, and the educative and preventive power of such labours cannot be over-estimated. The central office of the society is at 7, Harpur Street, London, W.C.

### News from India.—South Travancore.

(COMMUNICATED.)

MEDICAL missionaries have a hard work in carrying on the fight against disease, ignorance, and superstition amongst native populations, but it is labour not without encouragement, and signs are not wanting that real progress is being made, in spite of many disappointments and much practice of the virtue of patience.

In South Travancore the Medical Mission works from fourteen centres, dealing with a population of 837,000. At each centre there is a hospital or dispensary, with a medical missionary in charge. The past year has seen such a hospital established at Martandam, containing two wards, for eight patients each, consulting, dispensing, and waiting rooms, and a small isolation ward. Mr. Yacob Yesudasen, the missionary in charge, has saved much expense, and greatly aided in the erection of the hospital, by personally superintending the building work, and collecting the necessary timber from neighbouring landowners. At Neilikakuri and Karakonam temporary buildings are being replaced by permanent ones. A maternity ward at Neyoor has been completed during the past year, and opened for patients. The needless suffering and loss of life amongst native lying-in patients is terrible, and it is very satisfactory to feel that European methods of treatment are gradually overcoming prejudice, and gaining increasing confidence.

More nurses have now been taken into the Neyoor Hospital, where there are now five in training under Miss MacDonnell, who gives them systematic teaching in general nursing, and especially in obstetric work. These trained Christian nurses are in ever-increasing demand among the better class of patients in their own homes, which is very encouraging. It is exceedingly difficult under ordinary circumstances to get medical orders carried out with regularity, for the native mind is governed by many small superstitions, such as an objection to give medicine on a cloudy and overcast day, which renders successful treatment of out-patients anything but an easy matter.

The mission has lost a good friend this year in the death of Mr. Rama Row, the late Dewan, whose help was always generously at the service of charity. His name will be perpetuated in the "Rama Row Mission Hospital" at Nedungolam, and the cot called after, and endowed by, him at Neyoor. One of his last acts was to arrange for the legal transfer of land given by him to the Mission.

At the suggestion of Dr. Neve, at the Mission hospitals bags filled with sawdust have been adopted as the best form of absorbent dressing. Soaked in 1–2,000 corrosive sublimate and dried in the sun they make a dressing reliable surgically, and very economical. For an under-dressing gauze is prepared from the thin unglazed muslin of the country, and answers its purpose admirably. Gifts in kind are very welcome from friends in England, especially in the form of toys for the little ones, linen and cotton rags, remnants of bright-coloured prints, unbleached calico, and turkey-red for quilts, huckaback towels, or dusters. All such contributions are most useful.

### Holidays and Health.

[Readers of The Hospital in need of information about health resorts at home or abroad, or desirous of aid in forming holiday plans, are invited to send queries to Editor, 428, Strand, W.C. (marked "Travel" on outside of envelope), which will be answered under this section.]

### NEWQUAY AND TINTAGEL.

Cornwall is not so far off as we fancy it is, thanks to the quick trains run by the Great Western, and few places within such an easy distance of London offer so thorough a change to the weary worker.

Newquay is on the north coast of Cornwall, and has consequently quite a different climate from Falmouth and Penzance, both of which places are usually too enervating to be pleasant summer resorts.

The journey to Newquay is delightfully simple. Through carriages run from Paddington, and as the holiday-seeker can arrive soon after six o'clock there is plenty of time to look round for suitable lodgings if they have not already been secured. Excellent accommodation is to be had, and the terms are decidedly moderate in the older part of the town, a little beyond the new and pretty lodging-houses by the station. There is also a capital and most comfortable boarding house (of which the editor has the address), which may be preferred to lodgings by those who do not care to have the trouble of housekeeping when out for a holiday; and as the owner of it is always delighted to provide sandwiches for those who wish to remain out all day, the inconvenience of having to be in for a fixed mid-day meal is quite obviated.

Newquay is a quaint little old-world place, consisting of one long street; niggers and trippers are unknown, and so are brass bands and cabs, the place of the last named being supplied by two burly fishermen who own a truck and a pony, and so monopolise the carrying trade of the place.

Few places possess more lovely or more varied natural beauties; at high tide huge green Atlantic breakers come surging and crashing in apparently close under one's feet, and at ebb tide there are long stretches of very yellow sand, delightfully firm to walk on. The many-coloured cliffs are quite a feature of the place, some of them reaching to a height of between three and four hundred feet. These cliffs are in several places completely honeycombed with caverns, about many of which wonderful tales of wrecking and smuggling are told; one of these caverns, the Tea Cavern, reaches inland for nearly three-quarters of a mile; it was formerly much used by smugglers, and so secure were its hiding-places that the Preventive men have been known to search it in vain, although they were certain that a whole ship's cargo of tea was stored in it.

The Cathedral Cave is a marvellously symmetrical hollow, so evenly vaulted and pillared that it is difficult to believe that man had no hand in its development.

Another cave on Porth Island has a hole in its roof, through which, at high tide, the spray bursts with a loud report, and ascends in a column to a height of twenty or thirty feet, taking the unwary lounger a good deal by surprise, for on the surface of the island all that appears is a small and innocent-looking crack or crevice, and this too at some distance from the sea. These caverns, with several others equally noted, form some of the Newquay sights.

The air is bracing without being boisterous, and the joy of "resting the tired eyeballs on the sea" is there especially great, for the colouring is unlike anything else in England, it is so vivid and varied, so entirely different from the sad-coloured North Sea or the muddy Channel. The intensely yellow sand is said to give the water its vivid green hue, and this green becomes the purest blue before it reaches the horizon, making sketches of it look somewhat garish, and of the "tea-tray" order.

There are many places of interest in the immediate neighbourhood, and within an easy walk are several fine old churches, which contain good carving and quaint epitaphs, and often remarkably perfect stone coffins. Everything is so different from what it is elsewhere, even the stiles having a construction peculiar to themselves. They are made by laying half a dozen great blocks of hewn granite side by side at an interval of about a foot, and over these gridiron-like arrangements human beings step, but they say cattle cannot.

The hedges, too, are unique, and are very lovely during the spring and summer; every flower of which one has ever heard is there in abundance, and all of them larger and more brilliant than usual. But the peculiarity of these hedges is that one walks on the top of them! They are in reality dykes, between five and seven feet high, with the path running along the top, both sides of the dyke being covered with the delicious smelling flowers.

From Newquay to Tintagel, with its crowd of poetic associations, is a pleasant drive; a coach goes there and back daily, and allows plenty of time for the visitor to inspect the remains of King Arthur's Castle. The natural position of this castle is a grand and imposing one, for it stands on a steep and rocky island, which is connected with the mainland by a wooden bridge. The ascent of the rock is somewhat perilous, in spite of the late rector having cut steps and placed a handrail up the rock to render the ascent easier. St. Juliet's Chapel is easily made out, and enough remains of the ruins of the castle to let us see what an extensive building it must once have been, but since the time of Henry VII. it has been allowed to fall into ruins.

Of King Arthur's original castle nothing of course remains, the natural objects only are the same as they were when the "blameless King" and his knights gazed upon them. Even to the utterly practical busy worker there is a halo of romance about everything; the entire place indeed seems steeped in it, and passages from Tennyson's "Idylls" come crowding in on our memory, until we can quite picture the King sallying forth with some of his knights to "smoke some hive of turbulent bees" that were working mischief in his realm on the mainland.

The narrow strip of water that separates the rock from the land is of a curiously inky blackness, and as it is of great depth it is rather awe-inspiring as it swirls and boils below, making visions of a false step not enticing.

Along the cliffs to Boscastle is a grand and wild walk, and Tintagel itself has plenty to interest those who care to spend a few days there. It possesses two comfortable little inns, where one is feasted on Cornish cream at every meal. Indeed, everywhere throughout the duchy the visitor is regaled on this delicacy, and even those who usually have to avoid the cream of the everyday world can enjoy quantities of this clotted or "clouted" variety. A Cornish breakfast, indeed, is considered to fall far short of perfection if it does not end with heather honey, fresh raspberries, and clotted cream.

In all ways Cornwall is a thorough change from the usual seaside places; even the people are different—tall, and with dark, swarthy complexions. They have quite the appearance of the traditional ancient Britons before the Saxon blending turned us into a fair-haired, fresh-complexioned race.

### Where to Go.

Royal British Nurses' Association.—The secretary requests us to state that the quarterly meeting of the General Council will be held on Friday, July 10th, at five p.m., at the offices of the Association, 17, Old Cavendish Street, W.

Bovril (Limited), 63, Bath Street, City Road.—The Bovril Company are arranging to hold a reception of nurses, "in and around London," on Tuesday, July 28th, at their warehouse in City Road. Lord Playfair will receive the visitors, and give them an address in the course of the afternoon. There will be tea and fruit, and the processes and exhibits at the factory will be on view. Tickets will be sent to nurses in due course.

### A Book and its Story.

"THE WORLD AND A MAN." BY Z.Z.

"Z.Z." is known to the literary world through his "Drama in Dutch." In his present contribution* to the world of letters he shows himself not only a matured writer, but a matured thinker. And yet his volume before us for review is prepared in an adroit manner to defy criticism, for in his short foreword the author declares his book to be above the charge of an "aim to accomplish anything." It is a book without a purpose, he assures us; whatever was in the writer's mind when he penned "The World and a Man," he leaves upon his reader's mind anything but a negative impression as to the outcome of certain social problems—problems which are all the more baffling of human solution when handled in the masterly spirit which "Z.Z." brings to bear upon them. The story, which is divided into three parts, headed respectively "Idealism," "Transition," and "Materialism," is the history of a young man with high-flown ideas and purposes, whose soul warred in indignation against his world, and against whose warring the world, in its turn, took revenge.

Luke Merrit was a young London citizen, who did not take life easily. His circumstances, even so far as mundane matters go, did not content him; he drudged on a stool in the office of his uncle, a London merchant, but his position did not seem to better itself. The young man spent his salary on such books as fostered his intellectual learnings; and his spare time he passed at Socialist meetings. He made converts of the two youngest and worst-paid clerks in the counting-house. This was but a limited success, measured by the daring of his words. But failure to convince others only resulted, we are told, in the strengthening of his own convictions. "And at length, as the result of intense morbid dwelling on the social system and of incessant analysis of its constitution, he could not taste or touch any of the products of labour without a mental picture of the corresponding labourers at work. If he poked the fire, immediately a realistic picture of miners digging, digging, toiling half-naked, rose before him. He took his walks to the swing of the pickaxe, to the rhythym of roaring machinery: the bitter cry of the unemployed, mounting meaningly above the din of factories and cities, rang in his ears. His own inner unrest was as the unrest of the oppressed multitude, as he contemplated the pent-up forces that were gathering to sweep all before them." Listeners he found, but not many, to his diatribes against the present order of society, the daughter of a certain conservative lawyer being among those who listened. At the Williams' house, a dingy house in an old-fashioned square in Bloomsbury, Luke Merrit had an established welcome. Here, over the chessboard, the aspiring young Socialist made the acquaintance of the lawyer's daughter Minnie, who accorded him the listening we have just referred to. It was Luke's evident earnestness that had first induced the girl to give him her attention, but soon she found herself absorbed. His plea for oppressed mankind touched her sympathies. So two years passed by, and Luke Merrit found himself in love. Hand in hand he and Minnie were to work out the world's salvation—for life in future meant life with Minnie. A sweet year raced by—a year of more reading, more discussions, more kisses, more dreams. "At the end both were steeped in communism, in social forethought, in social freedom, in every kind of literature that piled contempt on traditional institutions." And whilst their elders were making the best of the world as it was, these two were pulling it to pieces, resetting it together, "raging at the inability of people in general to distinguish between that which was ordered by the Supreme Power and that which was ordered by man."

About this time Luke's uncle raised him to the dignity of country traveller. And time went on, crowded with toil, movement, thought, argument, and alternative spasms of bitterness and hope. It was three years since Minnie and he had plighted their troth, though the prospect of any consummation of their hopes seemed as far off as ever.

Mention has already been made of the views the two shared of social institutions, and now the question of putting one of these theories into practice was first mooted by the man. This having been all along more or less present in his mind as an inevitable consequence of their common point of view, it had never arisen in conversation between them. A letter from her betrothed setting forth these views somewhat startled Minnie. Here Luke said "that marriage, as she must know, meant to him the voluntary union of two human beings in perfect sympathy; that love alone sanctified such union before the Supreme Being."

The girl had caught much of Luke's heroic vanity, of his spirit of Quixotism. Fascinated, she played with the idea. "There would be something grand, original, daring, defiant in carrying it into effect." Luke Merrit had now rid himself of his uncle's care and set up a line of trade for himself. Minnie had turned her back on her parents' home at his bidding, and joined Luke, who earned enough to keep the small home going. In spite of serious difficulties, he was fairly successful, "and with the renewed success came increased strength of mind, so that he was able to take up his studies again for an hour or so each evening. It was good for him, however, that, apart from his reading, he had little time for speculative thought, for his health and spirits improved as his attention was centred on his material affairs." Now and again, if his mind did revert to the old grooves, the puzzles and problems which thronged upon him were excluded by the urgent necessity of giving an undivided attention to the business of life.

Here, Part II. of the book—"Transition"—commences; the young man's life is passing onwards, if not upwards—and it reaches a culminating point in Part III., "Materialism." In the final chapters of the book "Z.Z.'s" hero is shown us in another light.

His programme has not been carried out, he has not reformed mankind, he has not earned any real livelihood, he has not established a home. For the first item he has not had sufficient free time—for the second, he has met with failure, for the third the sharer of his home has departed, having discovered that she loves another man with whom, later, she enters into marriage.

Thus, without much of the bombast of his early years, a man whose earnest beliefs have given way to a lasting cynicism, Luke Merrit returns to his uncle's home. "And as he looked back now, his whole past stretched before him, one long, pitiful piece of self-deception, it was apparent," to quote the author's words, "to his present dispassionate vision, how little his ideals had really influenced his actions. Those actions, he saw clearly, had been mainly determined by his mere instincts and impulses. His ideals had not, to any great extent, saved him from following those impulses; they had served but to make him half-hearted, perplexed, hesitant." And then we leave Luke Merrit picturing himself back again in his uncle's house, sitting over the port after a comfortable dinner, in the old, stately dining-room, and his uncle saying "Didn't we always tell you you would get over all your nonsense?" and he, himself, smiling back assent. And in the end he, Luke, would make a rich marriage and settle down a grave, respectable citizen, who accepted all the conventions and the traditions. Perhaps, too, he would become a Churchman; perhaps an alderman.

* "The World and a Man." By Z.Z. (London: William Heinemann. 1896.)

### Everybody's Opinion.

[Correspondence on all subjects is invited, but we cannot in any way be responsible for the opinions expressed by our correspondents. No communications can be entertained if the name and address of the correspondent is not given, or unless one side of the paper only be written on.]

### PRIVATE NURSES' FEES.

"A PRIVATE NURSE" writes: You are kind enough to ask for the opinion of "private nurses working on their own account" as to the question of payment raised by your correspondent in last week's HOSPITAL, and I am glad to have an opportunity of saying what I think on the matter. My experience of doctors is that they do not help a nurse to good paying cases as a rule when they find she can afford to lower her fees. I have never known a private nurse who would take 10s. 6d. a-week, for to do this she must have private means, or a home for which she has not to pay whilst at a case (as most nurses have to do); therefore, she is independent of her earnings, and it will be immaterial to her if she loses a better case. Again, such a precedent would be exceedingly injurious to other nurses, for it is quite a custom now with a certain class—who could afford to pay two guineas a week—to try and get a nurse to lower her fee, sometimes to one guinea, "because they really cannot afford such an expense." Where such a payment as 10s. 6d. a week could only be afforded I should consider the case would more properly belong to the district nurse. If a *bonâ fide* case of real poverty with serious illness came under my notice, and no district nurse were available, I would willingly give my services for nothing, but would not lower my fee to 10s. 6d. With regard to a doctor not charging the poor patient on the same scale as his wealthy ones, the cases do not appear to me to be parallel. The doctor has many cases, and is not out of pocket in attending a poor one; a nurse has but one, and in such circumstances, unless independent of her profession, would be certainly a loser.

### Appointments.

THE HOSPITAL OF ST. CROSS, RUGBY.—Miss Anna Beatrix Baillie has been appointed Matron at this hospital. She was trained at the General Infirmary, Gloucester, and at the London Hospital, where for the last three and a-half years she has held the responsible post of Sister of the Mellish Accident and Surgical Wards. Miss Baillie takes many good wishes with her to her new work from her fellow workers at the London Hospital, by whom she will be much missed.

ST. CATHERINE'S CONVALESCENT HOME, PENN, NEAR WOLVERHAMPTON.—Miss Edith Jordan has been appointed Matron at this home. She received her training at the Guest Hospital, Dudley, afterwards working as assistant nurse at the General Hospital, Wolverhampton, and then as private nurse in connection with the Nurses' Home, Malvern. Miss Jordan undertook holiday duty as matron at the St. Catherine's Home last year, and has now succeeded permanently to that post on the resignation of Miss Jury. Her testimonials are excellent, and we congratulate Miss Jordan upon evidently well-deserved promotion.

ST. MARK'S HOSPITAL, SALT LAKE CITY, UTAH.—Miss M. Mitchell (Sister Mary) has been elected Superintendent of the Nurses' Training School in connection with this hospital. Miss Mitchell is an Englishwoman, and a Nightingale nurse, and many old friends will be interested to hear of her appointment. After training in the Nightingale School Miss Mitchell had the charge of medical and surgical wards at the Royal South Hants Infirmary, and was for two years sister-in-charge of the Rotunda Auxiliary Hospital, Dublin. We cordially wish her all success in the future.

### Minor Appointments.

THE NEW INFIRMARY, ISLEWORTH, W.—Miss E. Rabarts has been appointed Night Superintendent at this infirmary. Miss Rabarts was trained at St. Marylebone Infirmary, and has been some years sister of a flat at St. Saviour's Infirmary, East Dulwich.

### Notes and Queries.

The contents of the Editor's Letter-box have now reached such unwieldy proportions that it has become necessary to establish a hard and fast rule regarding Answers to Correspondents. In future, all questions requiring replies will continue to be answered in this column without any fee. If an answer is required by letter, a fee of half-a-crown must be enclosed with the note containing the enquiry. We are always pleased to help our numerous correspondents to the fullest extent, and we can trust them to sympathise in the overwhelming amount of writing which makes the new rules a necessity. Every communication must be accompanied by the writer's name and address, otherwise it will receive no attention.

### Queries.

(93) *Madeira or Canary Isles.*—Please tell me something about Madeira and the Canary Isles as health resorts. Are they suitable for invalids during winter months?—*C. S.*

(94) *Paying Probationers.*—Would you kindly tell me which of the London hospitals take lady probationers for three months' training? Is the fee about one guinea a week?—*Emigrant.*

(95) *Lady Roberts' Nurses.*—Can you tell me how I can obtain particulars of Lady Roberts' Nursing Staff in India; also how should a nurse set about obtaining work in one of our hospitals abroad?—*B. H.*

(96) *Training.*—Would a monthly nurse, trained in that branch of nursing only, be likely to find permanent work in any hospital? Is there any hospital at which one could train for two years, receiving a salary, and does an infirmary-trained nurse rank as one fully trained?—*S. B. R.*

(97) *Advice Wanted.*—Can you recommend me any preparation to recover black hair, prematurely turned grey?—*Nurse Ellen.*

(98) *Quarterly Payment.*—Please tell me whether wages that are paid quarterly are reckoned from September 25th or 29th for the last quarter of the year?—*Sheffield.*

(99) *Chiropody.*—Can you tell me of a chiropodist in London?—*Anxious One.*

### Answers.

(93) *Madeira or Canary Islands* (*C. S.*).—Both Madeira and the Canary Islands are very favourite resorts for invalids during the winter months. Hotel accommodation is good. The principal hotel at Funchal, Madeira, is famed for its good management and fine situation. In the Canaries there are the Métropole and Catalina Hotels at Las Palmas, and Quiney's Hotel at Monte, a few miles inland. At Teneriffe the principal hotel is the Taoro, at Oratava. Here there is a resident lady nurse. Madeira is more popular for lung diseases, the Canaries for that as well as for sufferers from rheumatism, diseases of kidney, &c. The climate is delightful, the Canaries being the more bracing, with a warm, dry air, while in Madeira it is warm and moist. The mean winter temperature is at Oratava 63·8 (Fahrenheit), in Madeira 61·7. Dr. Victor Perez, the resident physician at Teneriffe, bears a high reputation in the island. In the Canaries, at any rate, the invalid element will not be found unpleasantly conspicuous.

(94) *Paying Probationers* (*Emigrant*).—Several of the London hospitals receive paying probationers for three months at a time. You will find full particulars in "How to Become a Nurse" (Scientific Press, 428, Strand). The fee is usually thirteen guineas for the three months. Write to the matrons of the London, Royal Free, Charing Cross Hospitals.

(95) *Lady Roberts' Nurses* (*B. H.*).—Lady Roberts chooses her own nurses privately; an introduction to her would, therefore, be the only way to obtain an appointment on her staff. With regard to hospitals abroad, your best plan would be to write to the matrons of the various hospitals, of which you will find a complete list in Burdett's "Hospitals and Charities."

(96) *Training* (*S. B. R.*).—You should go in for general hospital training, and there are hospitals both in London and the provinces where it is possible to obtain a two years' training. At most hospitals, however, paid probationers are not given a certificate under a term of three years. Read "How to Become a Nurse" (Scientific Press, 428, Strand). Twenty-three to twenty-five is the usual age at which probationers are entered into general hospitals, a little younger at children's hospitals. A nurse with a certificate from a good infirmary training school certainly "ranks as fully trained."

(97) *Advice Wanted* (*Nurse Ellen*).—You cannot "recover" hair once bleached. You could only dye it, and surely it is far better to keep your hair in a natural condition, even if it be prematurely grey. Grey or white hair, too, is, as a rule, far from unbecoming. There is always more or less danger in hair dyes, which frequently contain injurious ingredients.

(98) *Quarterly Payment* (*Sheffield*).—The date of quarterly payment of wages entirely depends upon the day of the month on which the service commenced. If, for instance, an engagement is entered upon on January 1st, payment is due on April 1st, July 1st, October 1st, and January 1st. Rent quarter days are March 25th, June 24th, September 29th, and December 25th.

(99) *Chiropody* (*Anxious One*).—Mrs. Stanton, 62, Mortimer Street, Cavendish Square, W., can be well recommended.

### Wants and Workers.

[THE attention of correspondents is directed to the fact that "Helps in Sickness and to Health" (Scientific Press, 428, Strand) will enable them promptly to find the most suitable accommodation for difficult or special cases.]

*District Nurse*, St. Agnes, Cornwall, writes to ask if any kind friend will send her left-off clothing, old sheets or blankets, boots or shoes, for the poor people in her parish. She will gladly pay carriage of any parcels.

## Figures and Tables

**Fig. 28. f1:**